# Tobamoviruses: Molecular Aspects and Resistance Regulation—A Special Issue Commemorating 125 Years of Research on Tobamoviruses

**DOI:** 10.3390/v17030296

**Published:** 2025-02-21

**Authors:** Peter Palukaitis, Alex M. Murphy

**Affiliations:** 1 Graduate School of Plant Protection and Quarantine, Jeonbuk National University, Jeonju 54896, Republic of Korea; 2 Department of Plant Sciences, University of Cambridge, Cambridge CB2 3EA, UK

## 1. Introduction

**Figure 1:**
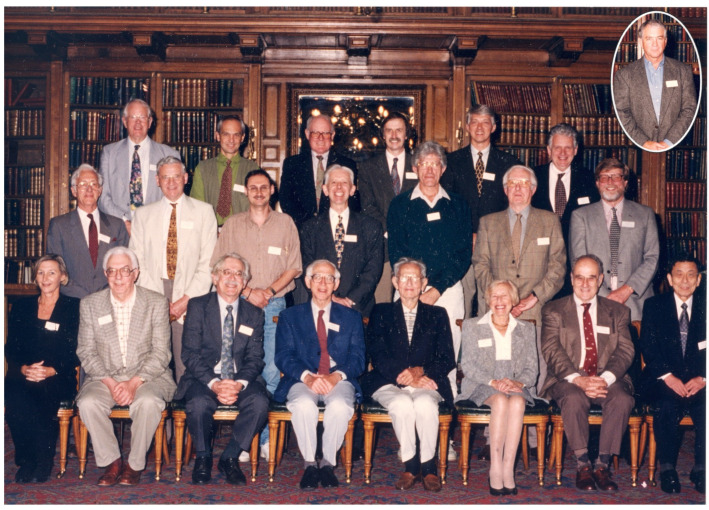
TMV 100 years meeting—participants. Left to right. Back row: Bryan D. Harrison, Tom H. Turpen, John G. Shaw, Gerald Stubbs, Roger N. Beachy, and Marc H.V. Van Regenmortel; middle row: Lute Bos, P.J.G. Butler, Vitaly Citovsky, Ken W. Buck, Adrian Gibbs, Fred Last, and T.M.A. Wilson; front row: Barbara Baker, Joseph G. Atabekov, Donald L.D. Caspar, Sir Aaron Klug, Heinz Fraenkel-Conrat, Bea Singer, Milton Zaitlin, and Yoshimi Okada. [Inset: W.O. Dawson]. Photo taken by Tom Geohegan, provided courtesy of P. Palukaitis.

This Special Issue of Viruses was commissioned in 2023 to celebrate 125 years of research on Tobamoviruses. Twenty-five years previously, in 1998, a two-day symposium was held at the Royal College of Physicians in Edinburgh, Scotland, to commemorate the 100th anniversary of research on tobacco mosaic virus, the first tobamovirus, from which the sigla (tobamo) was derived. The meeting, held on 7–8 August 1998, was organized by Bryan D. Harrison and T. Michael Wilson, both then belonging to the former Scottish Crop Research Institute (now the James Hutton Institute), with assistance from Fred Last (meetings convenor for the Royal Society of Edinburgh, to facilitate use of the venue), and sponsored by the Royal Society of Edinburgh, in association with the Royal Society, London, UK. The hosts and invited speakers of the symposium are shown in Figure 1. Most papers presented here were subsequently published [1,2,3,4,5,6,7,8,9,10,11,12,13,14,15,16,17,18,19], and a summary of the meeting presentations and discussion was published in *The Plant Cell* [20].

Since that meeting in 1998, the field has advanced apace, and despite there now being ~50 known tobamoviruses at the time of this writing, most of the basic molecular research has been conducted on three related tobamoviruses: tobacco mosaic virus (TMV), tomato mosaic virus (ToMV), and pepper mild mottle virus (PMMoV). Several Brassicaceae-infecting tobamoviruses have also been used for virus movement studies. The other tobamoviruses have largely been used for studies on evolution, host adaptation, and/or biosecurity topics, with the latter often focused on descriptions, methods of detection, ecology, and/or epidemiology. Some of those topics were covered in a previous Special Issue on Tobamoviruses, published in 2023 by *Viruses* [21]. This Special Issue features five review articles authored by leaders in the field, whose laboratories, in recent years, have been actively engaged in tobamovirus research.

## 2. Plant Immunity Against Tobamoviruses

The first article published in this Special Issue, by Xiyin Zheng, Yiqing Li, and Yule Li [22], reviewed the literature on plant immune responses against various tobamoviruses. The topics covered include components of the hierarchical zig-zag immune defense system (pattern-triggered immunity and effector-triggered immunity, and for the latter, the associated resistance (*R*) genes), RNA silencing (as well as viral RNA silencing suppressors), RNA decay, and autophagy. The authors also examined the roles of phytohormones and reactive oxygen species in mediating these defense responses, as well as the abilities of viruses to overcome a portion of these defense responses.

## 3. Obtaining the Tobamovirus Particle—Why and How? Purification Routes over Time and a New Customizable Approach

The second article, by Tim Wendlandt, Beate Britz, Tatjana Kleinow, Katharina Hipp, Fabian J. Eber, and Christina Wege [23], presented a history of the purification of TMV, and provided a brief history of the TMV work conducted in Germany, in particular in the 1940s and 1950s, at the beginning of the age of molecular biology. The authors also described the application of tobamoviruses for use in nanotechnology, and summarized investigations into the modifications needed to purify tobamoviruses altered on the surface of virus particles. As materials used in nanotechnology, such alterations affect the stability of virions and necessitate less severe methods for virion purification. This article was dedicated to the memory of Holger Jeske (1952–2022), scientific mentor, friend, collaborator, and/or encouraging discussion partner to the various authors.

## 4. Engineered Resistance to Tobamoviruses

The third article, by John P. Carr [24], reviewed the history of TMV and its early importance in agriculture, especially in tobacco, tomato, and chili pepper (along with ToMV and PMMoV), but which waned as resistance genes were found and deployed into various cultivars of these hosts. However, more recently, two other tobamoviruses have become major, global agricultural pathogens, viz. tomato brown rugose fruit virus (in tomato) and cucumber green mottle mosaic virus (in cucurbit species). The author also discussed various methods of control that have been developed using tobamoviruses, such as cross-protection and numerous biotechnological approaches, including transgenic viral protein-mediated protection, RNA silencing-mediated resistance, the topical application of double-stranded RNA (produced synthetically, in vitro, or inside bacteria), the introduction of resistance genes from other crops, and that of the CRISPR-Cas system to disable the host genes required for tobamovirus replication. Finally, the ongoing need to utilize the current approaches, as well as to develop new strategies to confer resistance to tobamoviruses, were discussed.

## 5. The Forgotten Tobamovirus Genes Encoding the 54 kDa Protein and the 4–6 kDa Proteins

The fourth article, by Peter Palukaitis, Masoud Akbarimotlagh, Sajad Astaraki, Masoud Shams-bakhsh, and Ju-Yeon Yoon [25], described the genome organization of tobamoviruses, vis à vis the phylogenetic analysis of 47 tobamoviruses, confirming and extending previously established relationships between phylogeny and the initial host family of isolation/limitation. The authors summarized earlier works on other proteins encoded by TMV and ToMV that are not usually mentioned in the literature, the first being a third subgenomic RNA encoding a 54 kDa protein in vitro that did not accumulate in planta, for which models were presented to explain such an effect, as well as the possible function of the 54 kDa protein. The second referred to a class of small proteins of 4–6 kDa, also encoded by TMV and ToMV, as well as a more recent work suggesting that these open reading frames (ORFs), either inside the movement protein (MP) ORF or overlapping both the MP ORF and the capsid protein ORF of most other tobamoviruses, may encode other small and larger proteins, now referred to as P6. The authors presented the possible roles for such small proteins in counter defense, based on the functions of small plant proteins.

## 6. Tobacco Mosaic Virus Movement: From Capsid Disassembly to Transport Through Plasmodesmata

The fifth article, by Amr Ibrahim, Nobumitsu Sasaki, James E. Schoelz, and Richard S. Nelson [26], reviewed the literature on the movement of TMV, and where known, on other tobamoviruses. This process starts with the disassembly of virus particles, leading to the translation of the viral genomic RNA and the interaction of the viral-encoded replication associated proteins with various host factors, which facilitated the formation of virus replication complexes (VRCs) and the synthesis of viral subgenomic RNAs. The latter were then translated to produce the MP and the capsid protein. A large body of work on the cellular biology of virus replication and movement was summarized and evaluated in the context of conflicting observations regarding the nature of the membranes associated with the VRCs, as well as the host and the viral-encoded proteins involved in the intracellular movement of the VRCs on different cytoskeleton components, with summary models presented. The research on the intercellular movement of TMV was examined and evaluated, describing the functions and cell biology of the TMV MP, as well as that on numerous host plasmodesmal factors that have been identified and characterized as being associated with and needed for viral movement between cells, also including a summary model.

## 7. Perspective

The above review articles exhibit that in the last 25 years, research on tobamovirus has thrived, and continues to make great strides, especially in areas such as cellular and molecular biology. The advent of new technologies such as nanotechnology has led to new applications for viruses in diverse areas, while novel methods for isolating the proteins involved in weak or transient associations between various viral protein–host protein complexes for proteomic analyses are also increasing our knowledge, enabling assessments of the nature of host proteins involved in resistance, pathology, virus replication, and movement. Research has also recently appeared involving protein–protein structure modeling and RNA–protein structure modeling, which will offer a greater understanding of the components involved in the above processes and their roles. Hopefully, the next 25 years will bear witness to even greater strides made in comprehending such processes and in controlling viruses.
